# New Developments in Views on Aging Research: Variability, Innovative Concepts, and Contextual Perspectives

**DOI:** 10.1093/geroni/igab046.1106

**Published:** 2021-12-17

**Authors:** Anna Kornadt, Hans-Werner Wahl, Susanne Wurm

## Abstract

Views on aging (VoA) such as attitudes toward own aging, awareness of aging or subjective age, have a large impact on outcomes related to positive development in later life. Recent research in this domain has focused on complex research designs and inter-systemic linkages at different levels. Indicators of short-term variability of VoA have increasingly been investigated, linking the respective findings with performance indicators, biomarkers, and trait-like data. In addition, bidirectional relationships of VoA and outcomes over time as well as data contextualizing VoA across historical time may offer new insights on the plasticity of VoA seen in bio-cultural co-construction. The symposium will showcase these recent trends with studies from the U.S. and Germany. First, Zhu and Neupert extend previous studies by linking established VoA indicators with future time perspective, all assessed by means of a daily diary study with 60-90 year-old adults. Kornadt et al. examined the variability of subjective age within a day and the relationship with trait subjective age and cortisol levels. Mejia et al. extend VoA to the area of subjective awareness of fall risks in daily life and links them with physical performance. Wettstein et al. investigate the bidirectional relationship of VoA indicators and perceived stress over time. Finally, we move from the micro to a macro-micro design in Wahl et al.’s presentation addressing historical change in VoA across 20 years in the Berlin Aging Study and in MIDUS. Susanne Wurm will discuss how different levels of VoA analysis will find better interlinkage in the future.

